# Macrophage-Mediated Inflammation and Disease: A Focus on the Lung

**DOI:** 10.1155/2012/140937

**Published:** 2012-12-12

**Authors:** Emily Gwyer Findlay, Tracy Hussell

**Affiliations:** ^1^MRC Centre for Inflammation Research, Queen's Medical Research Institute, University of Edinburgh, 47 Little France Crescent, Edinburgh EH16 4TJ, UK; ^2^Manchester Collaborative Centre for Inflammation Research (MCCIR), University of Manchester, Manchester M13 9PL, UK

## Abstract

The lung is exposed to a vast array of inhaled antigens, particulate matter, and pollution. Cells present in the airways must therefore be maintained in a generally suppressive phenotype so that excessive responses to nonserious irritants do not occur; these result in bystander damage to lung architecture, influx of immune cells to the airways, and consequent impairment of gas exchange. To this end, the resident cells of the lung, which are predominantly macrophages, are kept in a dampened state. However, on occasion the suppression fails and these macrophages overreact to antigenic challenge, resulting in release of inflammatory mediators, induction of death of lung epithelial cells, deposition of extracellular matrix, and development of immunopathology. In this paper, we discuss the mechanisms behind this macrophage-mediated pathology, in the context of a number of inflammatory pulmonary disorders.

## 1. Pulmonary Macrophage Populations

The distinct environment of the lung, with high oxygen tension [1] and constant exposure to inhaled antigen, both harmful and harmless, presents challenges for the immune cells which patrol the airways. The inhaled matter must mostly be ignored, in order to prevent overreaction and subsequent bystander tissue damage in response to nonserious challenges; such a response would fill the alveoli with immune cells and disrupt the delicate gas-exchange process. However, immune cells must be able to respond rapidly to a genuine threat and, once it is dealt with, resolve any resulting inflammation and remodel any damage to the lung tissue. This is a complex list of requirements and so it is not surprising that on occasion the balance between ignorance, response, and resolution tips in the wrong direction, resulting in immunopathology.

In the noninflamed airway, very few haematopoietic cells are present. Of the cells in the fluid from bronchoalveolar lavage (BAL) of naive tissue, alveolar macrophages (AM) (identified as CD11c^+^CD11b^−^MHC II^low^ autofluorescent cells) constitute >90% [2–4]. As the first cell type to encounter inhaled antigen, AMs are superb phagocytes, rapidly clearing bacteria from the airways [5]. They also help to maintain the dampened immune characteristics of the airways by producing IL-10 [6] and directly suppressing both dendritic cells (DC) and T cells [7–9]. After infection has resolved, they clear the cellular debris remaining [10] and aid in the remodelling of the lung parenchyma. The unique environment of the lung and the constraints on AM result in these pulmonary macrophage populations expressing different surface molecules to those elsewhere in the body; for example, AMs express high CD11c, owing to the high GM-CSF and surfactant protein D levels in the alveoli [11], which is not seen elsewhere, and which may aid the AM in their phagocytic function.

The second and third most common cell types in the airways (and the dominant immune cells in the lung tissue) are DC (CD11c^+^ MHC II^high^) and inflammatory monocytes (CD11c^−^MHCII^−^CD11b^+^) [3]; cells of lymphoid origin are sparse as monocyte-derived cells dominate. In addition to the resident steady-state lung populations, monocytes move in rapidly after the onset of inflammation. Both resident and immigrating macrophages are implicated in the development of pulmonary immunopathology.

## 2. Macrophages as Mediators of Lung Pathology

Macrophages are critical in the clearance of pulmonary pathogens [5, 12, 13]. However, in the balance between responding to dangerous inhaled pathogens and in maintaining a healthy airway free of immune cells, macrophages occasionally tip into immunopathology. To avoid this, AMs are kept in check by a variety of mediators in the lung. Surfactant proteins A and D bind to the negative regulator SIRP-*α* on the AM surface [1]; its signalling induces repression of activation and of their phagocytic function. Alveolar epithelial cells also produce IL-10 [14], which suppresses co-stimulatory molecule expression by the AM.

The accumulation of myeloid populations in the lung is firmly linked to the development of disease. During early influenza infection, airway epithelial cells produce CCL2 [15] (MCP-1), which attracts CCR2^+^ monocytes into the lung tissue from the blood [3]. Monocyte numbers peak on day 5 after influenza infection as the cells upregulate CD11c and MHC class II, before differentiating into either macrophages or monocyte-derived DC [3]. During this influenza infection, the majority of immigrating inflammatory cells are from CCR2^+^ monocytic parents [3]; mice lacking CCR2 not only have decreased accumulation of monocytes after influenza infection but also decreased mortality and lung damage [3, 16, 17], with CCR2^−/−^ mice exhibiting decreased lactate dehydrogenase (LDH, a measure of damaged and therefore leaky epithelium) in the BAL fluid. Ablation of CCR2 signalling also lessens lung damage in mouse models of pulmonary fibrosis and *Mycobacterium tuberculosis* [18, 19]. 

It is, therefore, clear that monocyte-derived cell types are critical mediators of inflammatory damage to lung tissue. In this paper, we discuss the mechanisms behind this damage in the context of a series of pulmonary inflammatory disorders.

## 3. Bacterial Infection and Cystic Fibrosis: Inflammatory Cytokine Storm

AMs are critical for the clearance of inhaled bacterial pathogens, which they achieve through phagocytosis, reactive oxygen species production, and the secretion of inflammatory cytokines and chemokines to attract other immune cells to the airways. However, the clearance of these pathogens comes at a heavy price as these inflammatory mediators can themselves lead to bystander damage of lung tissue.

In cystic fibrosis (CF), this inflammatory damage is a severe problem, with 85% of deaths as a result of persistent inflammation triggered by recurrent rounds of bacterial infection, clearance, inflammation, and remodelling [20–22]. AM switching from producing IL-10 to instead secreting a range of inflammatory cytokines (including TNF, IL-1*β*, IL-6, and IL-8) is well known to be key in the development of CF lung disease [23].

A recent study investigated this inflammatory cytokine production by AM in the response to *Burkholderia cenocepacia* and *Burkholderia multivorans*, which are key pathogens suffered by CF patients that initiate infections which result in overwhelming inflammation, cell death, and sepsis [24]. *B. cenocepacia* can infect AM as well as lung epithelial cells [25] and indeed both AMs of CF patients and of CFTR^−/−^ mice are more permissive to infection and persistence of the bacterium, with the mouse CFTR^−/−^ cells showing delayed phagolysosomal clearance compared to controls [26, 27].

Infection with *B. cenocepacia* results in overwhelming cytokine production by macrophages, in particular TNF [28], IL-8 [29], and IL-1*β* [2, 30]. This is a result of PI3K/Akt signalling inactivating GSK3*β*, a downstream repressor of NF-*κ*B, thus allowing enhanced NF-*κ*B activity, and consequent significantly higher cytokine production [26]. This macrophage production of IL-1*β* is enhanced in the absence of a functional CFTR channel [24] and is caspase-1 and TLR4-dependent. It is this hyperreaction by pulmonary macrophages which triggers the inflammatory storm and induces pathology and severe lung damage.

## 4. Influenza Infection: TRAIL and Death of Alveolar Epithelial Cells

In 2008, Lohmeyer and colleagues proposed an interesting new mechanism by which macrophages contribute to pathology during influenza infection [16]. It had been noted previously that patients with highly pathogenic influenza virus infection suffered widespread destruction of the respiratory epithelium [31, 32], but how this occurred was not clear. Lohmeyer's group showed that during infection of mice with PR/8 influenza, CCR2^+^ monocytes which were recruited to the lung and became exudate macrophages were mediating this cell death [16, 33].

**Figure 1:**
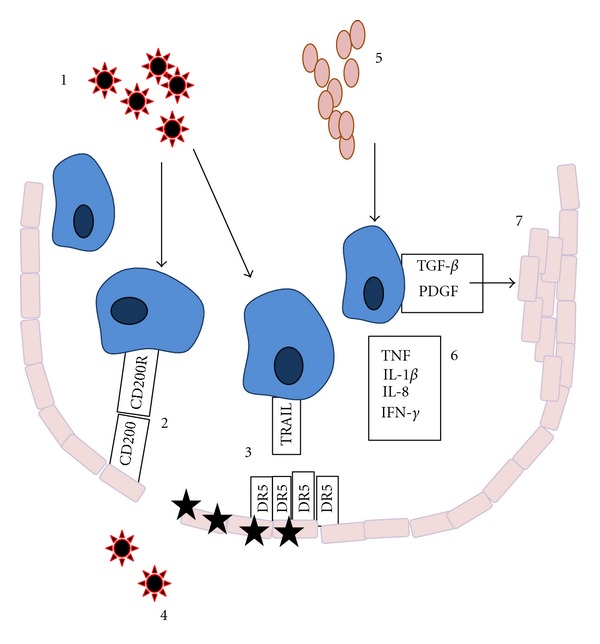
Alveolar macrophage-mediated disease in the lung. AMs are the dominant cell type in the uninfected airway. Following virus infection (1), AMs are restrained from overreacting and inducing bystander damage to tissues by the CD200R on their surface (2) which induces a negative signalling cascade. (3) Pandemic influenza strains induce the upregulation of TRAIL on the AM surface and of its receptor DR5 on the alveolar epithelial cells, which leads to increased apoptosis in the epithelium and consequent morbidity. Both the presence of TRAIL and the absence of CD200R can induce epithelial damage and spread of virus out of the alveolus (4). Bacterial infection (5) induces AM to produce inflammatory cytokines (6), leading to activation of bystander cells and tissue damage. AMs are also triggered to produce growth factors (7) which leads to epithelial hyperplasia, deposition of ECM and fibrosis.

On moving into the lung, CCR2-recruited exudate macrophages upregulate TRAIL (TNF-related apoptosis-inducing ligand), with mRNA levels fourfold higher than peripheral blood monocytes in the same infected mice [16]. Correspondingly, although all alveolar epithelial cells express a low level of DR5, the TRAIL receptor, it too was upregulated following influenza infection (Figure 1). Blockade of TRAIL on these cells resulted in lessened alveolar epithelial cell death and alveolar leakage, and improved survival. Interestingly, this TRAIL-induced death was seen in the highly pathogenic PR/8 but not the milder X31 infection, indicating this may be an additional mechanism by which pandemic flu strains damage the lung.

Following these interesting results, a more recent paper showed blockade of CCL2 following influenza infection led to an increase in epithelial damage [34], with this being explained by the reduced numbers of cells leaving the AEC more open to infection, ascribing a protective role only to AM, and indeed a role in repairing the influenza-infected epithelium following clearance of the infection. Indeed, anti-CCL2 treatment resulted in a decrease in hepatocyte growth factor (HGF) present in the lung [34]; HGF augments resolution and repair of damaged epithelium. In this paper, however, the virus used was the sublethal Aichi strain, supporting the theory that this AM-mediated cell death is only a factor in highly pathogenic strains.

## 5. Secondary Bacterial Infections: CD200 and CD200R

We [35] and others [36] have previously described the role of the CD200R on macrophages in the context of influenza infection. CD200 is expressed on airway epithelial cells, T cells, B cells, and some DC [35, 37–40], but has no intracellular signalling motif; its function is to bind to CD200R on myeloid-origin cells and induce a negative signalling cascade. AM express a high basal level of the receptor, allowing them to maintain a strong threshold for response in the normal lung, and prevent bystander tissue damage in the context of inhaled, but nonpathogenic, antigen. CD200^−/−^ mice develop severe immune-mediated lung damage and morbidity following influenza infection [35] as a result of the loss of this “dampening” of the pulmonary macrophage population (Figure 1).

More recent studies with these mice have linked the CD200/R pathway to the development of bacterial pneumonia following a primary influenza infection [41]. Secondary bacterial infections following influenza, particularly pandemic strains, are common and are responsible for sudden development of pneumonia. In the 1918 influenza pandemic, pneumococcal cultures could be grown from the majority of patients [42] and in the decades since then, the link between severe influenza-related pneumonia and secondary bacterial infections has grown stronger.

The role of macrophages and their negative regulation in an influenza—*Streptococcus pneumoniae* coinfection model was investigated [41]. In the absence of CD200R on AM, outgrowth of *S. pneumoniae*, sepsis and death were reduced. It was proposed that following an initial viral infection, an upregulation of CD200 on pulmonary immune cells and production of IL-10 means that the negative signals received by both alveolar and exudate macrophages are greatly increased. This is presumably to allow the lung a period of grace to clear dead cells and to remodel post-infection. However, the “immune rheostat” can swing too far towards repression, resulting in the lung macrophage populations not reacting to bacterial infection as they ought, and consequent outgrowth and sepsis [41]. As a majority of clinical influenza cases are thought to involve a secondary bacterial infection, this pathway is of significant therapeutic concern.

## 6. IPF: Production of Arginase and Proinflammatory Cytokines

Idiopathic pulmonary fibrosis (IPF) is a progressive interstitial lung disease, which is proposed to develop as a result of overexuberant remodelling following pulmonary epithelial damage [43], and which is characterised by chronic inflammation, alveolar epithelial hyperplasia, and deposition of extracellular matrix leading to development of a permanent “scar” [19, 44]. There has been much interest in the possible role of viruses in the development of the inflammation; in a mouse model, mice defective in IFN-*γ*R signalling are unable to clear murine herpesvirus infection, and the ensuing chronic infection leads to symptoms very similar to IPF [45, 46].

Recently, a role for macrophages has been delineated in this pathological process. In a model of IPF induced by intratracheal FITC deposition, lung damage was lymphocyte-independent and reduced in CCR2^−/−^ mice [19]. Macrophages are also dominant in the *γ*-herpesvirus infected mouse lung, and they localise to areas of epithelial hyperplasia and remodelling [47]. CCL2 and CCL3 are produced in the lung in the early stages of inflammatory fibrotic disease [47], attracting macrophages so that they can phagocytose debris, produce matrix metalloproteinases (MMPs) to alter the ECM degradation or alternatively produce ECM components themselves. These useful roles for macrophages are a result of their alternative activation; macrophages activated by Th2 cytokines upregulate a number of genes linked to wound healing, proliferation, and angiogenesis, including the secretion of a number of growth factors and of fibronectin (reviewed in [48]).

The mechanism by which macrophages are pathological in IPF is a result of this alternative activation. Lung fibrosis mediated by *γ*-herpesvirus is associated with recruitment of macrophages to the lung, their exposure to Th2 cytokines and subsequent alternative activation. Both the damaged lungs and the macrophages within stained positive for arginase 1, in both a mouse model and in patient samples [47]. AAMs have increased arginase activity; its role in converting arginine to ornithine, proline, and polyamine induces proliferation of fibroblasts, collagen production and, subsequently, the development of fibrosis [49]. 

In addition to arginase, macrophages are a critical factor in the initiation of fibrosis through their production of TNF, IL-6, IL-1*β*, and TGF*β* and of platelet-derived growth factor (PDGF) ([50], reviewed in [44]). These, in particular PDGF and TGF*β*, induce the proliferation of myofibroblasts, which secrete collagen [51]. Pulmonary macrophages also secrete MMPs, which degrade the extracellular matrix and so attract more immune cells to the site, perpetuating the inflammatory and remodelling cycle and exacerbating the formation of scar tissue.

## 7. Summary

In the absence of infection, cautiously reactive alveolar macrophages are the perfect guardians of the immune response in the lung, preventing overreaction to inhaled antigen and maintaining a generally “suppressive” environment. In addition, both alveolar and exudate macrophages are important in the early clearance of pathogens. However, following antigenic challenge, macrophages can tip from protection into immunopathology, with the best features that make them ideal to patrol the airways—cytokine and chemokine production, killing and phagocytosis of infected cells, clearance of debris, and remodelling of the lung—becoming damaging. The untethering of AM constraints and their resulting inflammatory cytokine storm in response to bacterial and viral infection can lead to development of epithelial cell death and consequent septic spread of bacteria to the blood, inappropriate migration of other cell types into the lung, clogging of the airways, deposition of ECM, and dysregulated repair of the damaged tissue. It is for these reasons that the constraining of AM responses during disease remains an attractive therapeutic target.

## Abbreviations

AM: Alveolar macrophages
AAM: Alternatively activated macrophages
BAL: Bronchoalveolar lavage
CF: Cystic fibrosis
DC: Dendritic cells
GMCSF: Granulocyte/macrophage colony stimulating factor
HGF: Hepatocyte growth factor
IPF: Idiopathic pulmonary fibrosis
PDGF: Platelet-derived growth factor
MMP: Matrix metalloproteinase
TNF: Tumour necrosis factor
TRAIL: TNF-related apoptosis-inducing ligand.
